# Malignant Pleural Mesothelioma (MPM) Presenting as Hydropneumothorax

**DOI:** 10.7759/cureus.41243

**Published:** 2023-07-01

**Authors:** Masami Kuramochi, Takuma Muraoka, Mayumi Shinonaga, Haruo Ohtani, Setsuo Kuraoka

**Affiliations:** 1 Thoracic Surgery, Mito Saiseikai General Hospital, Mito, JPN; 2 Pathology, Mito Saiseikai General Hospital, Mito, JPN

**Keywords:** malignant pleural mesothelioma, hydropneumothorax, pleural effusion, pneumothorax, immune checkpoint inhibitor

## Abstract

An 86-year-old man presented with bilateral lower limb edema and was found to have hydropneumothorax on chest radiography. CT revealed a substantial pleural effusion and plaques. The patient had a history of working in a stone workshop, but the extent of asbestos exposure remained unknown. Thoracic drainage and subsequent thoracoscopic surgery confirmed the presence of biphasic malignant mesothelioma through pathological examination. Hydropneumothorax as a presentation of malignant pleural mesothelioma (MPM) is rare, with only a few similar cases reported. Remarkably, despite the coexistence of plural effusion and pneumothorax, the patient did not experience dyspnea. The examination also revealed tumor rupture and disruption of the pleura. Considering the possibility of MPM in patients with asymptomatic hydropneumothorax is essential for early diagnosis and appropriate management.

## Introduction

Malignant pleural mesothelioma (MPM) is an aggressive tumor originating from the mesothelial cells lining the pleural cavity and is commonly associated with asbestos exposure [1]. Typically, MPM manifests with symptoms such as dyspnea, cough, and chest pain, while hydropneumothorax is an infrequent finding for its detection. Limited reports have described cases of MPM presenting with hydropneumothorax [2-5]. This report presents a similar case and discusses the distinctive clinical characteristics. Despite the presence of pleural effusion and pneumothorax, the patient remained asymptomatic. This article explores the pathogenesis, challenges in diagnosis, and the importance of considering MPM in cases of asymptomatic hydropneumothorax. Early detection and comprehensive evaluation are critical for effectively managing this uncommon presentation of MPM.

## Case presentation

**Figure 1:**
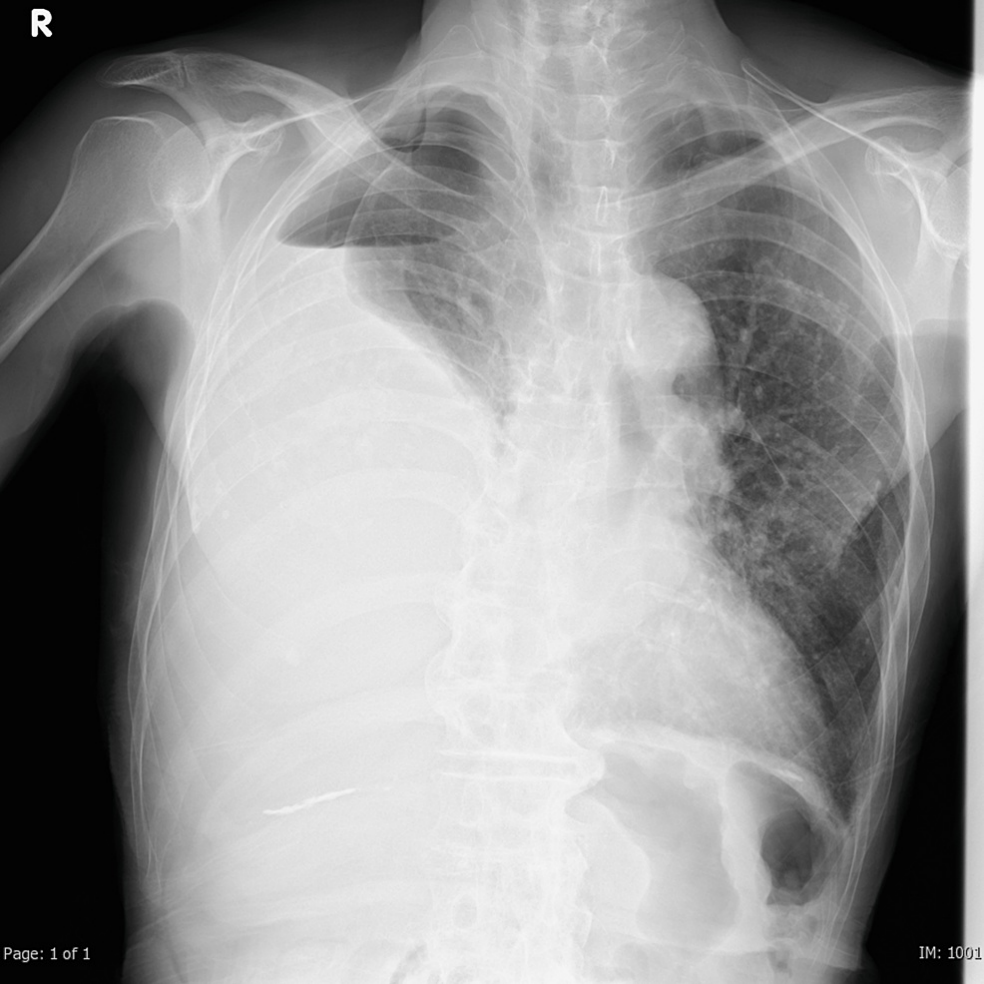
Chest radiography revealed reduced translucency and a pneumothorax in the right lung field.

**Figure 2:**
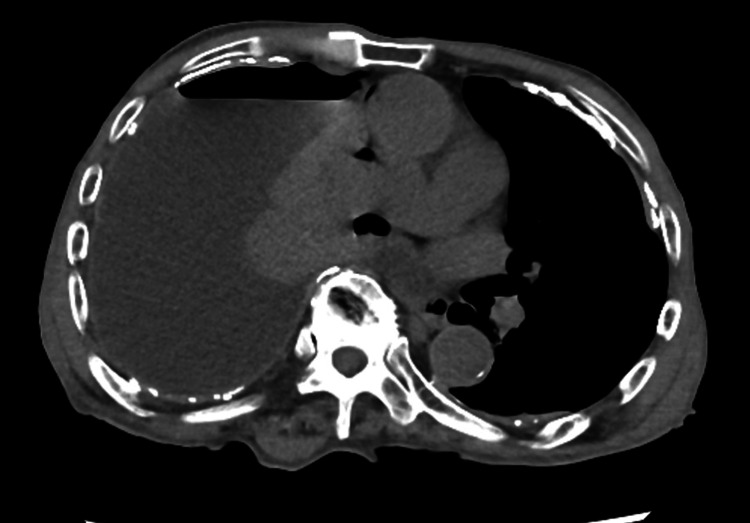
CT revealed a significant pleural effusion on the right side and multiple pleural plaques.

An 86-year-old man with a history of hypertension presented to a local clinic with bilateral lower limb edema. Chest radiography revealed a suspected right-sided hydropneumothorax, which prompted a referral to our department. The patient exhibited a body temperature of 36.8°C, and their SpO2 in ambient air was 96%. Chest radiography revealed reduced translucency and a pneumothorax in the right lung field (Figure 1). The echocardiogram revealed preserved cardiac function, and the lower extremity venous ultrasound did not indicate any abnormalities such as deep venous thrombosis. CT revealed a significant pleural effusion on the right side and multiple pleural plaques. No evident thickening or nodules were observed in the visceral or parietal pleura, and no apparent abnormalities were noted within the lung parenchyma (Figure 2). The patient had a work history in a stone workshop, but the extent of asbestos exposure was unknown. There was no history of tuberculosis, and the patient never had smoking habit. Thoracic drainage was performed on the same day, resulting in the extraction of approximately 1,500 mL of serous pleural effusion. Acid-fast bacillus staining of the pleural fluid was negative, and cytological examination did not reveal atypical cells. Due to persistent air leakage and a daily drainage volume of 50-80 mL, thoracoscopic surgery was performed seven days after drainage. 

**Figure 3:**
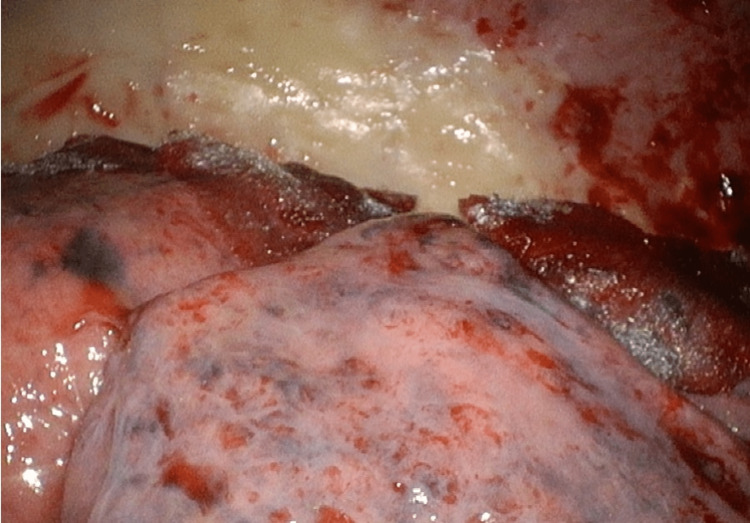
Operative finding revealed thickened parietal pleura with plaque and fragile visceral pleura. After partial resection of the lung, the resection site was covered with a polyglycolic acid sheet and fibrin glue.

**Figure 4:**
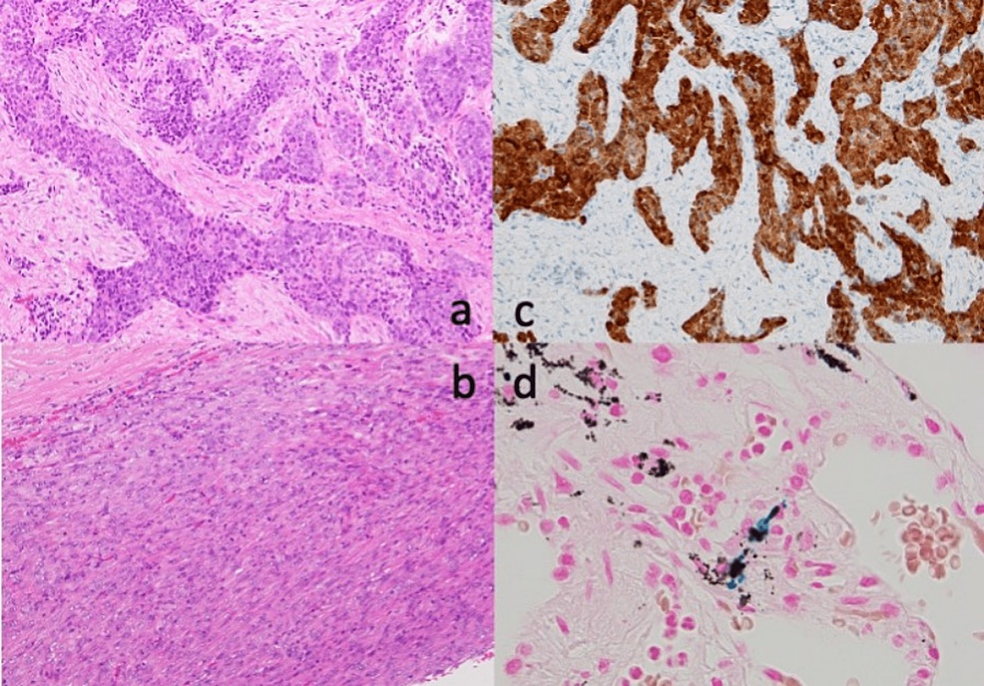
Hematoxylin-eosin staining revealed an epithelioid (a) and sarcomatoid (b) lesion of mesothelioma. Carletinin immunostaining was positive (c). Asbestos bodies were identified with Berlin blue staining (d).

Intraoperatively, we observed thickening of the parietal pleura with plaque formation, whereas the visceral pleura appeared fragile, susceptible to damage, and prone to bleeding. Artificial lung inflation revealed several lung fistulas on the surface of the visceral pleura. The identified leakage sites were excised and covered with a polyglycolic acid sheet and fibrin glue (Figure 3). Partial resection of the parietal pleura was performed, followed by pathological examination. On the second postoperative day, 10 Klinische Einheit of OK-432 was injected into the pleural cavity for pleurodesis, and the drain was removed on the third postoperative day. Pathological examination revealed marked fibrosis in both the parietal and visceral pleura, with proliferating atypical epithelial cells forming glandular structures and a dense proliferation of spindle cells (Figure 4a,b). Immunohistochemistry indicated the positivity of tumor cells (particularly the epithelial tumor cells) for calretinin, WT-1, D2-40, CK AE1/AE3, and CK5/6 (Figure 4c). Based on these findings, the diagnosis of biphasic malignant mesothelioma was confirmed. Tumor cells focally invaded 1.0 mm deep into the lung parenchyma. Berlin blue staining also detected asbestos bodies (Figure 4d). The tumor was staged as pT2cN0cM0 stage IA (Union for International Cancer Control-Tumor, Node, Metastasis classification version 8).

Considering the patient's advanced age and their inability to tolerate highly invasive surgeries, such as extrapleural pneumonectomy, or aggressive cytotoxic chemotherapy, treatment with immune checkpoint inhibitors (ICIs, Ipilimumab + Nivolumab) was continued. To date, the patient remains alive and has maintained stability for eight months following treatment with ICIs.

## Discussion

Malignant pleural mesothelioma is a challenging-to-treat tumor originating from the pleural mesothelial cells and is often associated with asbestos exposure [1]. Since MPMs originate from the parietal pleura and typically result in symptoms such as dyspnea, cough, and chest pain, hydropneumothorax is rarely the primary motive for its detection. In our literature search encompassing studies published from 2000 to the present, including our case, only 18 cases have been reported [2-5].

Furthermore, despite the coexistence of pleural effusion and pneumothorax, the patient did not complain of dyspnea. Kono et al. reported a case of MPM with hydropneumothorax in which the patient remained asymptomatic and was diagnosed during a routine medical checkup. They speculated that the slow accumulation of pleural effusion might have contributed to the lack of respiratory distress awareness and that lung collapse due to preexisting pleural effusion could account for the absence of symptoms even when pneumothorax occurred [6]. Our case exhibits similarities to theirs. In our case, chest radiography at the time of the initial examination revealed pneumothorax; however, a substantial amount of pleural effusion was already present, resulting in advanced lung collapse.

Mannes et al. described the pathogenesis of pneumothorax in MPM as the rupture of a necrotic tumor or disruption of the bra and brevis due to the tumor's obstruction of peripheral bronchi [7]. Although our patient displayed no cystic changes or gross neoplastic lesions, microscopic examination revealed a diffuse tumor extension to the pleural surface, indicating disruption of the visceral pleura by the tumor. Ema et al. reported a similar case and noted that this finding represents relatively early-stage MPM that can occur even in the absence of tumor invasion into the lung parenchyma [4].

Initially, when encountering hydropneumothorax, the differential diagnosis primarily focused on diseases involving lung parenchymal dysfunction, such as pneumonia, pulmonary tuberculosis, and lung cancer, rather than malignant pleural diseases. However, surgery was performed both for treating pneumothorax and for diagnostic purposes, leading to the identification of MPM. In cases of asymptomatic hydropneumothorax, the possibility of MPM should be considered, necessitating thorough investigations.

## Conclusions

This case report highlights a rare presentation of MPM as hydropneumothorax. Despite the presence of hydropneumothorax, the patient remained asymptomatic due to the preexisting pleural effusion and resultant lung collapse. Microscopic examination revealed tumor extension to the pleural surface, indicating MPM. Early diagnosis and comprehensive investigation of MPM are essential in cases of asymptomatic hydropneumothorax.
